# Unexpected Consequences of Noise-Induced Hearing Loss: Impaired Hippocampal Neurogenesis, Memory, and Stress

**DOI:** 10.3389/fnint.2022.871223

**Published:** 2022-05-10

**Authors:** Senthilvelan Manohar, Guang-Di Chen, Dalian Ding, Lijie Liu, Jian Wang, Yu-Chen Chen, Lin Chen, Richard Salvi

**Affiliations:** ^1^Center for Hearing and Deafness, University at Buffalo, Buffalo, NY, United States; ^2^Department of Physiology, Medical College, Southeast University, Nanjing, China; ^3^School of Communication Science and Disorders, Dalhousie University, Halifax, NS, Canada; ^4^Department of Radiology, Nanjing First Hospital, Nanjing Medical University, Nanjing, China; ^5^Auditory Research Laboratory, University of Science and Technology of China, Hefei, China

**Keywords:** hippocampus, neurogenesis, noise-induced hearing loss, memory, spatial navigation, stress, glucocorticoid receptor (GCR)

## Abstract

Noise-induced hearing loss (NIHL), caused by direct damage to the cochlea, reduces the flow of auditory information to the central nervous system, depriving higher order structures, such as the hippocampus with vital sensory information needed to carry out complex, higher order functions. Although the hippocampus lies outside the classical auditory pathway, it nevertheless receives acoustic information that influence its activity. Here we review recent results that illustrate how NIHL and other types of cochlear hearing loss disrupt hippocampal function. The hippocampus, which continues to generate new neurons (neurogenesis) in adulthood, plays an important role in spatial navigation, memory, and emotion. The hippocampus, which contains place cells that respond when a subject enters a specific location in the environment, integrates information from multiple sensory systems, including the auditory system, to develop cognitive spatial maps to aid in navigation. Acute exposure to intense noise disrupts the place-specific firing patterns of hippocampal neurons, “spatially disorienting” the cells for days. More traumatic sound exposures that result in permanent NIHL chronically suppresses cell proliferation and neurogenesis in the hippocampus; these structural changes are associated with long-term spatial memory deficits. Hippocampal neurons, which contain numerous glucocorticoid hormone receptors, are part of a complex feedback network connected to the hypothalamic-pituitary (HPA) axis. Chronic exposure to intense intermittent noise results in prolonged stress which can cause a persistent increase in corticosterone, a rodent stress hormone known to suppress neurogenesis. In contrast, a single intense noise exposure sufficient to cause permanent hearing loss produces only a transient increase in corticosterone hormone. Although basal corticosterone levels return to normal after the noise exposure, glucocorticoid receptors (GRs) in the hippocampus remain chronically elevated. Thus, NIHL disrupts negative feedback from the hippocampus to the HPA axis which regulates the release of corticosterone. Preclinical studies suggest that the noise-induced changes in hippocampal place cells, neurogenesis, spatial memory, and glucocorticoid receptors may be ameliorated by therapeutic interventions that reduce oxidative stress and inflammation. These experimental results may provide new insights on why hearing loss is a risk factor for cognitive decline and suggest methods for preventing this decline.

## Introduction

Intense noise primarily damages the sensory hair cells and spiral ganglion neurons; their destruction reduces the flow of acoustic information to numerous structures within the central auditory pathway as well as other parts of the brain that utilize auditory information to carry out complex processes such as formulating an emotional response to a baby’s cry, reacting viscerally when called to supper or exiting a train when your station is announced over the intercom. In order to respond effectively in these situations, the neural activity relayed through the ascending auditory pathway must be quickly and continuously integrated with information being processed in other parts of the brain such as those involved with motor control, cognition, emotion, and memory.

The hippocampus is generally considered as a structure involved in the formation of new memories, cognitive maps, and spatial navigation. Although the hippocampus lies outside the classical auditory pathway, it nevertheless responds to sound (Bickford and Wear, 1995; Moxon et al., 1999; Moita et al., 2003) and other sensory stimuli (Tamura et al., 1992; Cooper et al., 1998; Levy et al., 2004; Martin et al., 2007; Zheng et al., 2010; Gener et al., 2013). Consequently, severe noise-induced hearing loss (NIHL) would be expected to deprive the hippocampus of auditory information, for example, remembering a sequence of telephone numbers or series of instructions on how to exit a building. In the past decade, there has been growing interest in understanding how hearing loss affects the hippocampus, much of this motivated by clinical studies showing that blast wave-induced hearing loss is associated with memory and cognitive impairments as well as epidemiological studies suggesting that hearing loss significantly increases the risk of developing dementia (Lin et al., 2011a). How hearing loss disrupts memory and cognitive function is a major unanswered question with enormous clinical implications (Slade et al., 2020; Johnson et al., 2021). To provide insights on how NIHL could disrupts hippocampal function, the following section will briefly review some of the structural and functional characteristics of the hippocampus associated with auditory processing.

## Hippocampus Overview

**Figure 1:**
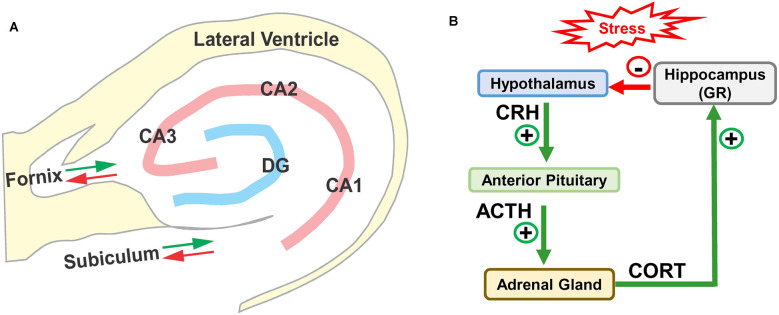
**(A)** Schematic of hippocampus and surrounding lateral ventricle. Three subdivisions of the rodent Cornu Ammonis (CA1, CA2, and CA3) surrounding the dentate gyrus (DG). Only the major afferent and efferent pathways (red/green arrows) through the subiculum and fornix are shown. **(B)** Schematic of hypothalamic-pituitary adrenal (HPA) axis with hippocampus. Stress stimulates the release (+) of corticotropic releasing hormone (CRH) from the hypothalamus, which binds to receptors in anterior pituitary causing the release (+) of adrenocorticotropic hormone (ACTH) which stimulates the release of corticosterone (CORT). CORT binds to glucocorticoid receptors (GRs) in the hippocampus, which provides negative feedback (−) to the hypothalamus suppressing the release of CRH, ACTH, and CORT.

The hippocampus is located in the medial portion of the temporal lobe adjacent to the inferior horn of the lateral ventricle (Lavenex, 2012; Wible, 2013; Fogwe et al., 2021). The two major components of the hippocampus are the dentate gyrus and the hippocampus proper, or Cornu Ammonis (CA) with three subdivisions in rodents, CA1, CA2, and CA3 (Figure 1A). The CA, shaped like a ram’s horn, wraps around dentate gyrus. The main afferent and efferent fibers in the hippocampus travel together in two major bundles, the fornix and subiculum. The fornix relays information between the hippocampus and multiple brain regions, principally the septal nuclei, preoptic nuclei, striatum, orbital cortex, cingulate cortex, thalamus, and mammillary body. Fibers in the subiculum relay information between the hippocampus and the entorhinal cortex and amygdala, which in turn connect to other areas such as the cingulate, olfactory bulb, and orbital cortex. Many of these structures form reciprocal connections with the hippocampus. Information from the entorhinal cortex is relayed through the performant pathway to the dentate gyrus. The dentate gyrus, in turn, transmits this information through mossy fibers to CA3 where it is relayed by Schaffer collaterals to neurons in CA1. Information from CA3 and CA1 is subsequently relayed through the fornix, fimbria, and subiculum to other regions of the brain. The output of the CA1 region of the hippocampus can also be directly relayed to the entorhinal cortex.

Pyramidal cells in the hippocampus receive glutamatergic excitatory inputs on numerous spines located on the apical dendritic shaft. The apical dendrites are oriented perpendicular to the pyramidal cells, whose somas are aligned in a thin layer along the CA axis. This stereotypic orientation causes the extracellular currents from individual neurons to summate and generate large field potentials. The primary axon of most pyramidal cells connects to neurons in the cerebral cortex, but collateral side branches emerging from the pyramidal cells form excitatory synapses on basket cell interneurons, which in turn synapse back onto the pyramidal cells. When activated, basket cells release the inhibitory neurotransmitter, gamma aminobutyric acid (GABA), generating recurrent negative feedback inhibition that dampens the activity of the pyramidal cells. Neurological conditions that reduce recurrent inhibition from the basket cells can lead to hyperactivity in pyramidal cells resulting in epileptic seizures and hippocampal sclerosis (Arellano et al., 2004; Cossart et al., 2005).

The hippocampus, considered part of the limbic system, forms connections with regions of the brain involved with emotion such as the amygdala, hypothalamus, and mammillary body (Miller and O’Callaghan, 2005; Cui et al., 2013; Kim et al., 2015). The hippocampus also contributes to the formation of new memories, cognitive maps, and spatial navigation (Moscovitch et al., 2005; Hartley et al., 2014; Ekstrom et al., 2017). The hippocampus contains place cells that respond vigorously when an animal moves into or through a specific place in the environment often in a specific direction. Place-specific neural firing has been observed in both pyramidal cells in CA neurons and granule cells in the dentate gyrus (Harvey et al., 2009; Bartsch et al., 2010; Mizuseki et al., 2011; Schmidt et al., 2012). These findings led to the hypothesis that hippocampal place cells are used to construct cognitive maps of the environment (O’Keefe, 1991; Krupic et al., 2018). In line with this view, patients with hippocampal lesions often suffer from amnesia and have difficulty on spatial navigation tasks and remembering where they have been (Banta Lavenex et al., 2014; Schoberl et al., 2019).

### Sensory Inputs to Hippocampus

The ability to spatially navigate through the environment to find food in a remote location requires an ongoing stream of multisensory information that can be compared against a cognitive map of the surroundings. Spatial navigation is believed to rely on three sources of information (Ravassard et al., 2013). These include visual cues from distant objects (O’Keefe, 1991), self-motion perceptual information (Gothard et al., 1996; Pastalkova et al., 2008; e.g., vestibular, proprioceptive), and information gleaned from other sensory systems (e.g., auditory, somatosensory, olfactory; Gener et al., 2013; Geva-Sagiv et al., 2015; Schinazi et al., 2016). The relative importance of these navigational cues varies with the nature of the task and the subject’s innate capabilities. In a brightly illuminated room, a rodent traveling through an eight arm radial maze to locate food in the northeast arm of the maze could use visual cues, together with odor, somatosensory, and auditory cues to navigate to the correct location. Although the visual acuity of rodents is poorer than that of primates (Prusky et al., 2000; Cruz-Martin and Huberman, 2012), they nevertheless use visual cues together with other forms of sensory information to remember where food can be found on subsequent searches of the maze.

Rats, however, are nocturnal animals and on a dark night, visual cues would be greatly reduced. Consequently, olfactory, somatosensory, and auditory cues would take on greater significance for navigating to the correct location. The sensory cues employed in spatial navigation also depend on the innate abilities of the species. Echo locating bats flying on a dark night and searching for its home in a dark cave would rely heavily on echolocation using auditory processing skills to return home.

### Neurogenesis, Memory, and Spatial Navigation

Neurogenesis refers to a process in which new neurons are generated from stem cells in the adult brain. Neurogenesis has been well established in the subventricular zone and hippocampus of adult rodents and non-human primates (Cinini et al., 2014), but in humans the evidence remains controversial (Boldrini et al., 2018; Sorrells et al., 2018; Kumar et al., 2019). The hippocampus in the rodent brain contains a stem cell niche. Approximately 9,000 new cells are born in the hippocampus of a young rat each day; most of these differentiate into neurons, migrate, and establish functional connections with other cells within a neural network (Cameron et al., 1993; Hastings and Gould, 1999; van Praag et al., 2002; Kuhn et al., 2018). There is a growing body of evidence linking hippocampal neurogenesis to the formation and retention of new memories related to spatial navigation, recognition, and declarative memory (Snyder et al., 2005; Aimone et al., 2006; Opitz, 2014; Bird, 2017). Aging, chronic stress, excess alcohol consumption, and cranial irradiation suppress neurogenesis, induce apoptosis and disrupts the formation of hippocampal dependent memories (Shors et al., 2002; Lucassen et al., 2006; Nixon, 2006; Warner-Schmidt and Duman, 2006; Winocur et al., 2006; Kubera et al., 2011). Conversely, antidepressant drugs, glucocorticoid antagonists, and exercise tend to enhance neurogenesis and improve memory (Encinas et al., 2006; Mayer et al., 2006; Oomen et al., 2007; Blackmore et al., 2009; ElBeltagy et al., 2010).

### Hippocampus and Stress

The hippocampus is especially vulnerable to several forms of trauma including chronic stress (Sapolsky, 1986; McEwen, 1994; Royo et al., 2006). High levels of stress activate the hypothalamic-pituitary-adrenal (HPA) axis (Figure 1B); this stimulates the release of corticotrophin-releasing hormone (CRH) from the hypothalamus, which in turn promotes the release of adrenocorticotrophic hormone (ACTH) from the pituitary gland. ACTH binds to receptors on cells in the adrenal gland, which leads to the release of corticosterone (CORT), a stress hormone in rodents (cortisol in humans). The released CORT crosses the blood-brain barrier into bloodstream where it can bind to the high affinity mineralocorticoid receptors (MRs) and low affinity glucocorticoid receptors (GRs). Under normal conditions, low-levels of CORT mainly binds to and activates MRs (see Ogita et al., 2012; Mifsud and Reul, 2018), but during periods of stress, CORT increases to high enough levels that it activates GRs. GRs are expressed on cells throughout the brain, but are heavily expressed on cells in the hippocampus (de Kloet et al., 1993; de Kloet and Meijer, 2019). The hippocampus is believed to be especially vulnerable to stress because it contains one of the highest densities of GRs in the brain (Joels et al., 2018). Indeed, high levels of CORT suppress hippocampal neurogenesis by hyperphosphorylating huntingtin, reducing brain derived neurotrophic factor (BDNF; see Agasse et al., 2020) and inducing dendritic atrophy on hippocampal pyramidal neurons. These negative effects are prevented by GR antagonists (Cameron and Gould, 1994; Magarinos and McEwen, 1995; Mayer et al., 2006; Warner-Schmidt and Duman, 2006; Morales-Medina et al., 2009).

### Auditory Inputs and Hippocampal Function

Auditory stimuli, such as a fire alarm, could affect the hippocampus indirectly by stimulating the release of CORT, or alternatively by stimulating the release of neurotransmitters from sound-sensitive neurons, such as those in the amygdala or septum, that project to neurons in the hippocampus (McDonald, 1998; Janak and Tye, 2015; Xiao et al., 2018) or from the lemniscal portion of the auditory pathway (Bickford et al., 1993; Moxon et al., 1999). Auditory evoked responses have been recorded from the dentate gyrus and CA of the hippocampus; the latency of the first peak of the evoked response in rats is approximately 30 ms, about 15 ms longer than the response from the inferior colliculus (Hall and Borbely, 1970; Jirsa et al., 1992). The threshold for eliciting a neural response from the hippocampus is roughly 25 to 35 dB higher than in the inferior colliculus. Of potential interest is the fact that the response amplitude from hippocampus can be enhanced by high-dose salicylate, an ototoxic drug that depresses the neural output of the cochlea (Chen et al., 2014). The early portion of the sound-evoked hippocampal response is largely abolished by destruction of the entorhinal cortex, which relays information to the hippocampus through the subiculum and perforant pathway (Deadwyler et al., 1981). Hippocampal neurons exhibit a range of specialized responses to sounds; some neurons exhibit directional sensitive responses while others respond to changes in frequency, intensity, tempo, and duration (Brown and Buchwald, 1973; Sakurai, 2002; Ruusuvirta et al., 2010; Geva-Sagiv et al., 2016). These acoustic features could be used to construct declarative memories as well as spatial and non-spatial cognitive maps.

Neural activity in the hippocampus is modified by auditory experience, especially sounds with biological significance (Deadwyler et al., 1981; Moita et al., 2003). After rats are trained on an operant auditory discrimination task, granule cells in the dentate gyrus acquire the “ability to distinguish” between two different auditory tokens by responding more robustly to positively reinforced sounds vs. unreinforced/negative stimuli. Hippocampal neurons acquire this preferential response to auditory stimulation through positive reinforcement (Deadwyler et al., 1981; Foster et al., 1988). Destruction of the perforant pathway, which relays auditory information from the entorhinal cortex to the hippocampus, largely abolishes neural responses to both positive and negative sounds (i.e., a non-selective effect). In contrast, lesions of the septal pathway impair auditory discrimination by reducing the difference in neural response magnitude to positive vs. negative auditory tokens (i.e., a selective effect). Thus, the septal pathway appears to relay information about the positive and negative attributes of the conditioned auditory stimulus (Foster et al., 1988).

Hippocampal neurons show evidence of both auditory working memory and reference memory. This is illustrated by a study in which rats were trained to discriminate between pairs of tones based on their temporal order vs. the similarity or difference in the pitch of the tones. To assess working memory, rats were trained to make a Go response if the current tone was different from the preceding tone and not respond (No-Go) if the current tone was the same as the previous tone (Sakurai, 1994). Alternatively, reference memory was assessed by training rats to make a Go response if the two sequential stimuli were both high-tones and to make a No-Go response if the two sequential tones were both low-tones (Sakurai, 1994). After training, some hippocampal neurons preferentially responded (i.e., produced more spike discharges or probability of firing to one task than the other) on the working memory task; others preferentially responded on the reference memory task and some responded on both the working memory and reference memory tasks. After operant reinforcement training, hippocampal neurons “formed memories” on how to differentially respond on an auditory working memory task vs. an auditory reference memory task (Sakurai, 1993, 1998).

Hippocampal place cells preferentially fire action potentials when an animal enters a specific location in its environment. The specificity and reliability of place cell firing is affected by information gleaned from external and internal sensory cues acquired by navigating through the environment on multiple occasions. Place cell performance is often evaluated in rodents using an eight-arm radial maze with food bait placed in one or more arms of the maze. While traversing through the maze, olfactory and somatosensory systems provide useful proximal cues while vision provides distal information (Quirk et al., 1990; Markus et al., 1994; Gener et al., 2013; Geva-Sagiv et al., 2016). However, in echolocating bats flying about in the dark, hippocampal place cells create an auditory map-like representation of a physical space using cues gained from echolocation (Geva-Sagiv et al., 2015).

Besides preferential response to physical place, hippocampal neurons are also able to create non-physical maps along a continuous auditory dimension such as sound frequency (Aronov et al., 2017). After rats were trained to physically change sound frequency by manipulating a joystick, the firing of hippocampal neurons increased as the rat shifted stimulus frequency in the direction of the target frequency; this occurred independent of other factors. Hippocampal neural firing occurred around discrete frequency fields only when the rat performed the task, whereas the same neurons did not respond when the same frequency was presented alone outside the situational environment. These non-spatial auditory frequency-fields often overlapped spatial navigation place-fields suggesting a common hippocampal mechanism not only for coding spatial navigation but also other non-spatial cognitive tasks such as remembering the correct sequence of sounds as in a melody.

Because the hippocampus is considered important for memory storage, it may come as no surprise that human functional imaging studies have implicated the hippocampus in storage of complex auditory information such as auditory hallucinations (Silbersweig et al., 1995; Takebayashi et al., 2002; Suzuki et al., 2003; Lefebvre et al., 2016) and simpler sounds such as the buzzing or ringing of the phantom sound of tinnitus (Lockwood et al., 1998; Chen et al., 2015). The phantom sound of tinnitus and musical hallucination often emerge following NIHL (Humes et al., 2006; Yankaskas, 2013) and other forms of acquired hearing loss (Rosanski and Rosen, 1952; Hammeke et al., 1983; Aizenberg et al., 1991; Tanriverdi et al., 2001).

## Noise-Induced Hearing Loss

### Permanent NIHL, Decreased Neurogenesis, and Memory Deficits

Recent epidemiological studies indicate that hearing loss is a risk factor for dementia and cognitive decline (Lin et al., 2011b; Deal et al., 2017; Su et al., 2017), suggesting the possible involvement of the hippocampus. Moreover, combat personnel exposed to intense blasts not only develop hearing loss (Cave et al., 2007), but also memory and/or other cognitive impairments (Belanger et al., 2009). It is unclear if these cognitive impairments result from the hearing loss *per se* or other factors such as the direct traumatic effect of the blasts on the brain as suggested by animal studies showing blast-induced neuropathology and tau protein expression in the hippocampus (Säljö et al., 2009; Sajja et al., 2015).

**Figure 2:**
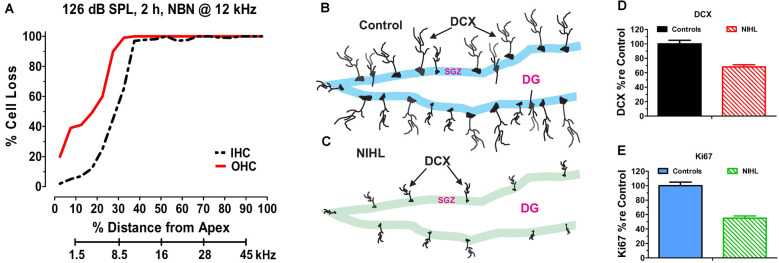
Noise-inducedhearing loss suppresses hippocampal cell proliferation andneurogenesis. **(A)** Cochleogram showing massive loss of outerhair cells (OHC) and inner hair cells (IHC) in the noise-exposed cochlear several months after a 2-h unilateral exposure to narrowband noise (NBN) centered at 12 kHz and presented at 126 dB SPL. Percent cell loss plotted as function of percent distance from the apex of the cochlear. Cochlear place related to frequency using rat tonotopic map on lower abscissa. **(B)** Schematic of dentate gyrus (DG) of hippocampus from normal control showing immunolabeled doublecortin (DCX) soma in the subgranular zone (SGZ); note extensive immunolabeled processes emanating from soma. **(C)** Schematic of DG of hippocampus several months after a noised induced hearing loss (NIHL) showing immunolabeled DCX) soma in the subgranular zone (SGZ). Note reduced number of DCX soma and paucity of labeled processes in the NIHL hippocampus compared to normal control (panel **B**). **(D)** Schematic showing relative number (% re Control: percentage relative to control) of DCX labeled neurons in hippocampus of normal control rats (100%) and rats with noise-induced hearing loss (NIHL). **(E)** Schematic showing relative number (% re Control: percentage relative to control) of Ki67 labeled neurons in hippocampus of normal control rats (100%) and rats with noise-induced hearing loss (NIHL).

The hippocampus is a major site of neurogenesis in the adult brain (Kaplan and Bell, 1984; Eriksson et al., 1998; Snyder et al., 2009) and recent animal studies have shown that NIHL can chronically suppress hippocampal neurogenesis (Kraus et al., 2010; Liu et al., 2016; Manohar et al., 2020). A persistent decline in neurogenesis was first reported in 2010 after adult rats had been unilaterally exposed to intense continuous noise (2 h, 126 dB SPL, narrowband noise, 12 kHz) and evaluated several months post-exposure. The unilateral noise exposure destroyed virtually all outer hair cells (OHCs) and inner hair cells (IHCs) over the basal two-thirds of the cochlea (Figure 2A), but it did not damage the hair cells in the contralateral cochlea that had been protected with an ear plug (Kraus et al., 2010). Neurogenesis was evaluated several months after the exposure by labeling hippocampal brain sections from noise-exposed and control rats with doublecortin (DCX), a protein expressed in developing neural precursor cells (Brown et al., 2003). In normal controls, DCX-labeled soma were arranged in a band running along the subgranular zone of the dentate gyrus (Figure 2B) and an elaborate network of processes extended from the soma of these neurons. In noise-exposed rats, by contrast, the number of DCX-labeled somas was greatly reduced and few neural processes emanated from the somas of these neural precursors (Figure 2C). Although only one ear was noise-damaged, the number of DCX-positive cells was reduced in both the ipsilateral and contralateral dentate gyrus by ~30% (Figure 2D). Ki67 immunolabeling was used to assess the rate of hippocampal cell division at the time of sacrifice several months post-exposure (Scholzen and Gerdes, 2000). Ki67 immunolabeling was reduced by more than 50% in the subgranular zone of the ipsilateral and contralateral hippocampus (Figure 2E). These results suggested that cochlear hearing loss might result in long-term cognitive or spatial navigation deficits.

Subsequent experiments conducted in adult mice bilaterally exposed to broadband noise (123 dB SPL, 2 h) revealed significant cognitive impairments on the Morris Water Maze test several months post-exposure (Liu et al., 2016). Noise-exposed mice with significant permanent hearing loss and massive OHC loss in the basal half of the cochlea had significantly more difficulty learning the location of the hidden platform (i.e., working memory deficits). Several weeks later, the noise-exposed mice also had more difficulty remembering where the hidden platform had been previously located (i.e., reference memory deficits). The chronic working memory deficits and reference memory deficits were associated with a bilateral decline in hippocampal neurogenesis (~27%) and cell proliferation (~26%). Moreover, the learning and remembering deficits were positively correlated with the degree of hearing loss. At the time when the memory tests were performed (~3-months post-exposure), there was no evidence of long-term oxidative stress in the hippocampus. In addition, CORT hormone levels were normal ruling out stress as a causal factor. Similarly, postnatal mice exposed to intense noise near the onset of hearing not only suffered from severe hearing loss in adulthood, but also suffered from chronic spatial learning and memory deficits and decreased neurogenesis several months after the noise exposure (Tao et al., 2015). These results indicate that NIHL in early life is a risk factor for learning and memory deficits in later life.

A persistent reduction in hippocampal neurogenesis was also observed in adult rats exposed to three blast waves with a peak pressure of 188 dB SPL (Newman et al., 2015). The bilateral blast exposure produced hair cell lesions in both ears. Approximately 25% of the OHCs and IHCs were missing over much of the cochlea, but in the extreme base of the cochlea, hair cell losses exceeded 85%. Hippocampal neurogenesis, assessed by DCX-labeling, was reduced by ~40% in the dentate gyrus several months after the exposure (Newman et al., 2015).

In subsequent experiments, working memory and reference memory were assessed approximately 3-months after rats were exposed to six blasts with a peak intensity of 185 dB peak SPL (Manohar et al., 2020). This bilateral exposure caused a severe hearing loss and greatly reduced the neural output of the cochlea as reflected in the compound action potential. Neurogenesis assessed by DCX-labeling was reduced by ~46%; this reduction was largely due to decreased cell proliferation rather than a decline in the proportion of new cells that differentiated into neurons, consistent with earlier results (Kraus et al., 2010). The blast-exposed rats performed as well as control rats learning the location of the hidden platform on the Morris Water Maze test (i.e., normal working memory). However, when retested several weeks later, the blast-exposed rats had difficulty remembering where the hidden platform had previously been located; results indicative of impaired memory consolidation (i.e., reference memory deficit). Thus, blast-wave induced hearing loss only caused a deficit in reference memory unlike previous work in mice in which working memory was also impaired (Liu et al., 2016). It has been suggested that active learning promotes the survival of new hippocampal neurons (Anderson et al., 2011; Shors et al., 2012; Curlik et al., 2013). However, in noise-exposed mice, active training on a Morris Water Maze task had minimal effect in promoting neuron survival (Liu et al., 2016).

### Acute Noise Exposure and Hippocampal Place Cells

**Figure 3:**
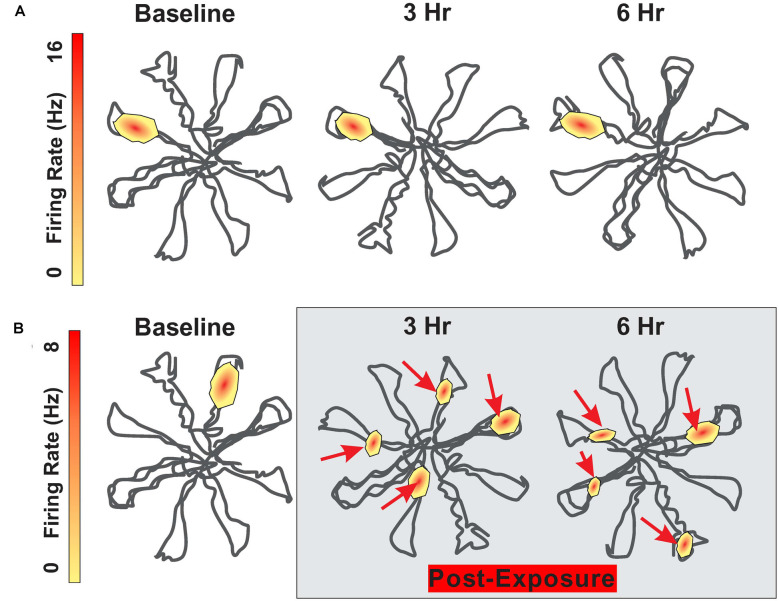
Schematic showing firing pattern of place cells in the hippocampus as rat navigates through an eight-arm radial maze. **(A)** Schematic showing place cell firing pattern within the radial maze; place at which the cell fires remain relatively stable between baseline and 3 h and 6 h later. **(B)** Schematic showing place cell firing pattern at baseline and then 3 h and 6 h following 30 min exposure to 104 dB SPL 4 kHz tone. Location of place cell firing locations drastically altered after the noise exposure. Maximum firing rate on upper and lower heat maps is 16 Hz and 8 Hz respectively.

The firing pattern of hippocampal place cells remain relatively stable for months as long as testing occurs in the same environment (Save et al., 2000; Agnihotri et al., 2004). Although visual, olfactory and somatosensory cues are considered the primary signals regulating place cell firing, auditory stimuli also appear to be important (Moita et al., 2003, 2004). The subtle contribution of auditory cues is illustrated when place cell fields are mapped out in an eight arm radial maze before and after an intense noise exposure. During baseline testing before the noise exposure, hippocampal place cells consistently fired at specific locations within the maze (Figure 3A). To determine the impact of intense noise exposure on place cell firing, rats were exposed for 30 min to a 104 dB SPL, 4 kHz tone (Goble et al., 2009). Place cell firing patterns were greatly disrupted after the noise exposure (Figure 3B). The original place field was shrunken and distorted and new place fields emerged. Instead of only firing at a specific location within the maze, cells began to respond at multiple locations within the maze. The disruptions of place field firing patterns began immediately after the noise exposure and persisted for at least 24 h. These results indicate that noise-induced changes in cochlear function results in unexpected changes in place-cell firing. Because cognitive function was not assessed, it is unclear if this noise exposure disrupted spatial navigation. It is unclear if the functional changes in place cell firing are temporary or permanent, but given that this noise exposure was not too severe, it seems likely that the place cell firing patterns might be restored as hearing loss recovers following the noise exposure.

### Acute Noise Exposure and Hippocampal Long-Term Potentiation

Neural circuits in the hippocampus exhibit different forms of synaptic plasticity. The most well studied form of synaptic plasticity is long-term potentiation (LTP), a prolonged increase in synaptic strength that occurs following repeated stimulation of a synapse such as the Schaffer-CA1 or perforant-dentate synapses. LTP has been considered a form of synaptic learning and memory. Repetitive auditory stimulation can influence hippocampal function (Angelucci et al., 2007; Deschaux et al., 2011; Kraus and Canlon, 2012; Nguyen et al., 2018) raising the possibility that intense noise exposure might disrupt hippocampal LTP and spatial navigation. Indeed, a 1-min exposure to high intensity sound stimulation (110 dB SPL), but not low intensity (80 dB SPL) stimulation disrupted hippocampal LTP for more than 24 h. However, neither the low or high intensity sounds failed to disrupt learning and memory performance assessed with the Morris water maze (de Deus et al., 2017). While short, intense noise exposures can easily disrupt hippocampal LTP (Cunha et al., 2018), noise-induced disruption of LTP does not appear to be a predictor of impaired spatial memory.

### Chronic Intermittent Noise Exposure, Neurogenesis, and Memory

**Figure 4:**
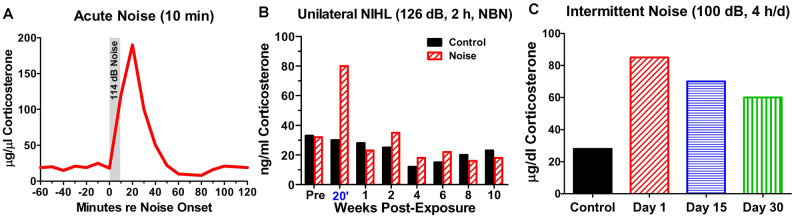
Noise-induced changes in corticosterone. **(A)** Schematic showing rapid rise and fall of serum corticosterone following a 10-min exposure to white noise presented at 114 dB SPL. **(B)** Schematic illustrating corticosterone levels measured over 12 weeks in sham control rats and rats exposed unilaterally for 2-h to 126 dB SPL narrowband noise (NBN) centered at 12 kHz. Corticosterone greatly elevated in noise group 20’ post-exposure, but levels decline to normal 1-week post-exposure. No significant difference in long-term basal corticosterone levels between control and noise-exposed group. **(C)** Schematic illustrating the rise in serum corticosterone following chronic intermittent noise presented at 100 dB SPL for 4-h/day over a period of 30 days. On day 1, corticosterone measured 30-min post-exposure while on day 15 and day 30, corticosterone was measured 24-h after the exposure.

From a mechanistic perspective, it may be important to distinguish between the chronic vs. acute effects of NIHL on the hippocampus. Stress hormones began to rise once noise levels reach 85 dB SPL and they continue to increase up to 110 dB, the highest intensity evaluated (Burow et al., 2005). However, this increase is normally temporary because CORT binds to GRs; this triggers negative feedback onto the hypothalamus depressing the release of CORT even if the stressful noise is continued (Dallman et al., 1992; Romero, 2004). Consequently, after a single, intense (114 dB SPL), short duration (10 min) noise exposure, CORT levels rise to a peak roughly 15 min after the start of the noise, but then return to baseline approximately 50 min after the noise is turned off (Windle et al., 2013; Figure 4A). In cases of very short duration, moderately intense noise exposure such as this, the hearing loss and cochlear damage are likely negligible.

**Figure 5:**
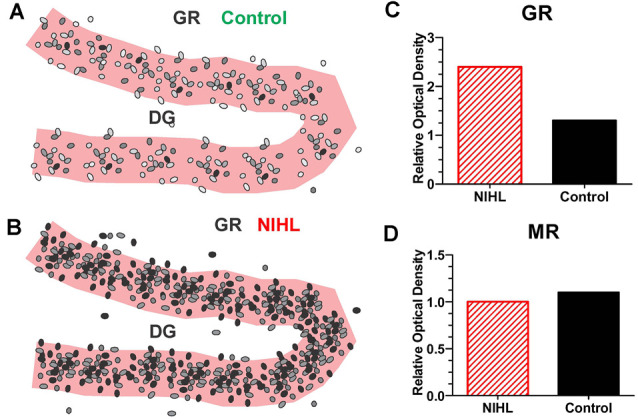
Severe NIHL alters glucocorticoid receptor (GR) expression in hippocampus. **(A)** Schematic of dentate gyrus (DG) in hippocampus showing immunolabeling of GR receptors (black, gray round, oval symbols schematically illustrate the relative intensity of immunolabeling.) in normal control. **(B)** Schematic of DG in hippocampus showing GR immunolabeling several months after induction of severe unilateral noise-induced hearing loss (NIHL; 126 dB SPL, 2 h, NBN centered at 12 kHz). **(C)** Schematic of relative optical density of GR immunolabeling in DG in rats with severe chronic NIHL compared to controls. **(D)** Schematic of relative optical density of mineralocorticoid receptor (MR) immunolabeling of rats with chronic NIHL compared to controls.

On the other hand, a single, very high intensity noise exposure lasting several hours (126 dB SPL, 2-h, narrowband noise at 12 kHz) is likely to cause significant hearing loss and hair cell damage (Hayes et al., 2019). Immediately after such a traumatic noise exposure CORT levels are transiently elevated, but after several days CORT levels recover to baseline and remain stable for weeks afterwards (Figure 4B). Although, basal CORT levels are normal, GRs are significantly upregulated in rats with NIHL compared to controls (Figures 5A,B); GR expression had increased roughly two-fold above normal (Figure 5C). In contrast, mineralocorticoid receptor (MR) expression levels in the NIHL rats were similar to controls (Figure 5D). The chronic upregulation of hippocampal GRs would likely disrupt negative feedback to the HPA axis, potentially contributing to a blunted response to stress (see Figure 1B).

However, if an intense noise (100 dB SPL) is repeatedly turned on (4-h) and off (20 h) for 30 days, CORT levels are chronically elevated. CORT levels are the highest 30 min after the noise is turned off on day1 (Figure 4C). CORT measurements obtained 24 h after the noise was turned off on day-15 and day-30 are only slightly lower than those obtained shortly after the noise was turned off on day-1 (Samson et al., 2007). Thus, the persistent elevation of CORT during chronic intermittent noise exposure could create a condition of unremitting stress leading to disruption of the HPA axis (Hebert and Lupien, 2007). The chronic stress to the HPA axis could only be alleviated if the subject habituates or adapts to the noise-induced stress response (Day et al., 2009; Masini et al., 2012). However, if chronic stress is unpredictable, it can chronically disrupt the HPA axis resulting in a blunted stress response, decreased neurogenesis, and increased inflammation (Algamal et al., 2018; Blossom et al., 2020; Parul et al., 2021).

The consequences of persistent exposure to intermittent noise are illustrated by a study in which rats were exposed for 15 days to 100 dB SPL noise for 2 h/day. The rats were then evaluated 15-days post-exposure when they exhibited a relatively mild NIHL (Shukla et al., 2019). CORT levels were significantly elevated several weeks after the intermittent noise exposure. Moreover, cell proliferation and neurogenesis were greatly reduced and spatial memory was impaired, consistent with the persistent increase in CORT after the exposure. However, if the rats were pretreated with an adenosine A2a receptor agonist, which exerts cytoprotective effects by increasing adenosine availability, the noise-induced hearing loss and disruptions of the hippocampus were greatly reduced (Fredholm, 2007; Wong et al., 2010). The protection of the hippocampus induced by this adenosine agonist is consistent with previous reports seen with other antioxidants and neuroprotective compounds (Herrera et al., 2003; Hinduja et al., 2015; Daulatzai, 2016).

### NIHL Accelerates Cognitive Decline in Alzheimer Models

With the worldwide increase in longevity, the prevalence of dementia and Alzheimer’s disease (AD) is expected to surge creating tremendous social and economic burdens (Alzheimer’s Association Report, 2020; Bennett et al., 2021; Farina et al., 2022). To understand the biological basis of the diseases, many rodent models of AD have been developed (Gotz et al., 2018) providing researcher with the opportunity to investigate the contribution of environmental factors such as NIHL in disease progression (Cui et al., 2015; Gai et al., 2017; Jafari et al., 2020; Paciello et al., 2021). In one study, triple transgenic AD mice were repeatedly noise exposed as young adults and cognitive and hippocampal function evaluated months afterwards, but before the expected time of cognitive decline. Prior noise exposure accelerated the onset of short-term and long-term memory decline in the AD mice. These early memory deficits were associated with abnormal synaptic function, increased neuroinflammation and enhanced tau protein expression in the hippocampus and were accompanied by noise-induced functional and morphological changes in the auditory cortex (Paciello et al., 2021).

Others have observed temporary (<7 days) increases in Aβ and amyloid precursor protein in the rat hippocampus after chronic noise stress (100 dB SPL, 4 h/day, 28-days; Cui et al., 2015). The short-lasting increases in the AD proteins are likely related to repeated daily stress induced by the 28-day intermittent noise exposure. If these daily stressful noise exposures were to continue over many months or years they could eventually lead to the chronic buildup of toxic AD proteins and long-term memory deficits. It has been known for many years that long-term exposure to moderate intensity intermittent noise can result in permanent NIHL (Johnson et al., 1976; Melnick, 1991); however, the preceding results suggest that prolonged exposure to unpredictable intermittent noise could also contribute to cognitive decline and dementia as suggested by epidemiological studies.

## Other Types of Peripheral Hearing Loss

### Reduced Neurogenesis With Conductive Hearing Loss and Ototoxicity

While this review has focused on NIHL as a disruptor of neurogenesis and memory, other types of peripheral hearing losses that deprive the hippocampus of auditory information might be expected to have similar effects. Indeed, clinical studies indicate that prolonged conductive hearing impairment in early life contributes to chronic auditory processing deficits, poorer social skills, language, reading, and cognitive deficits (Zinkus and Gottlieb, 1980; Reichman and Healey, 1983; Bidadi et al., 2008; Williams and Jacobs, 2009; Purcell et al., 2016). These findings suggest that chronic conductive hearing loss could negatively impact the hippocampus by reducing the flow of auditory information to the brain without damaging the sensorineural elements in the cochlea. Indeed, when auditory inputs to young mice were suppressed by surgically occluding one or both ear canals for 5 weeks, hippocampal cell proliferation and neurogenesis were suppressed and these effects were more severe when both ears were blocked (Kurioka et al., 2021). Unilateral conductive hearing loss suppressed neurogenesis bilaterally in the hippocampus, similar to the effects seen with unilateral NIHL (Kraus et al., 2010). Stress hormone levels were elevated 1 week after surgically occluding both ear canals raising the possibility that chronic stress was responsible for decreased neurogenesis. However, one argument against this view is that stress hormones are unlikely to remain elevated during the entire 5 week of ear canal occlusion because GRs in the hippocampus provide negative feedback to the hypothalamus that prevents the chronic release of stress hormones (Hayes et al., 2019). The validity of this hypothesis could be tested by regularly monitoring stress hormone levels over the period during which chronic ear canal blockade occurred.

Clinical reports suggest that temporary conductive hearing loss in early life, when the nervous system is rapidly developing, could contribute to permanent cognitive and memory deficits (Reichman and Healey, 1983; Williams and Jacobs, 2009). Support for this hypothesis comes from studies in which postnatal rats were subjected to a temporary bilateral conductive hearing loss. Hearing largely recovered when rats reached adulthood. Nevertheless, the rats with early-age temporary conductive hearing losses manifested significant deficits on working memory and reference memory tasks when they reached adulthood. These cognitive deficits were associated with reduced hippocampal cell proliferation, a decrease in hippocampal LTP and fewer hippocampal dendritic spines and post-synaptic densities (Zhao et al., 2018).

### Cisplatin Ototoxicity

Many drugs used clinical to treat cancer such as platinum-based antitumor drugs or life threatening bacterial diseases such as aminoglycoside antibiotics are ototoxic (Rybak, 1986; Arslan et al., 1999). These drug have long been known to cause permanent cochlear hearing loss by damaging the sensory hair cells, support cells, and spiral ganglion neurons in the cochlea. However, platinum based antitumor drugs such as cisplatin and carboplatin which block cell division could have potentially devastating effects on the hippocampus by suppressing cell proliferation and neurogenesis in the hippocampus. Cisplatin, one of the most widely used antineoplastic agent, has a number of well-known side effects including ototoxicity (Helson et al., 1978; Ravi et al., 1995), nephrotoxicity (Fillastre and Raguenez-Viotte, 1989), and neurotoxicity (Cavaletti et al., 1996). Other less well recognized complications include memory and attention impairments often referred to a “chemobrain” (Troy et al., 2000; Hede, 2008; Chiu et al., 2017). Cisplatin, which blocks cell division, crosses the blood-brain barrier (Nakagawa et al., 1996) and when administered *in vivo* to rodents robustly suppressed cell division and neural progenitors in the dentate gyrus. Cisplatin also damaged synapses, increased pro-apoptotic gene expression and enhanced cell death for at least 6 weeks following treatment (Dietrich et al., 2006; Andres et al., 2014; Manohar et al., 2014; Hinduja et al., 2015). Rats treated with high-dose cisplatin exhibited both learning and memory deficits on the Morris water maze test of spatial memory; these deficits were unlikely due to nonspecific health effects because swim speed and distance traveled in the cisplatin group did not differ from controls (Oz et al., 2015). These deficits were attributed to cisplatin’s neurotoxic effects on the hippocampus; however, it is possible that cisplatin-induced hearing loss is also a factor.

### Cyclodextrin Ototoxicity

Other ototoxic drugs such as the aminoglycoside antibiotics induce serious side effects such as nephrotoxicity, neurotoxicity, anemia, and thrombocytopenia (Snavely and Hodges, 1984; Prayle et al., 2010) making it difficult to disentangle the effect of cochlear hearing loss from more generalized effects on the central nervous system and hippocampus in particular. Unlike cisplatin and aminoglycoside antibiotics that are accompanied by numerous side effects, it may be possible to rapidly induce a hearing loss with minimal side effects with a single high dose of cyclodextrins (Crumling et al., 2017). 2-Hydroxypropyl-beta-cyclodextrin (HPβCD), which chelates cholesterol, is used to treat Niemann-Pick C1, a fatal neurological disorder caused by the intracellular buildup of lipids. High doses of HPβCD initially destroy the OHCs causing a 40 dB hearing loss (Liu et al., 2020). Approximately 6 weeks later the IHCs, organ of Corti, and spiral ganglion neurons degenerate resulting in a significant hearing loss and nearly total loss of OHCs and IHCs over most of the cochlea. Such lesions would deprive the central auditory pathway and hippocampus of nearly all auditory information. Approximately 4 months after treatment with 4,000 mg/kg of HPβCG, our preliminary studies revealed a massive reduction DCX immunolabeling in the dentate gyrus of the hippocampus. Because HPβCD has few side effect, these results suggest that massive cochlear damage may be sufficient to suppress neurogenesis. However, further research is needed to determine HPβCD-induced hearing loss that disrupts spatial learning and memory.

## Future Directions

### Preventing Cognitive Decline

If NIHL and other forms of peripheral hearing loss impair memory and increases the risk of dementia, then hearing restoration could conceivably slow or reverse these losses. Among elderly patients with profound postlingual deafness, only 25% had normal cognitive scores prior to cochlear implantation (Mosnier et al., 2015). However, 1 year after cochlear implantation, the percentage of subjects with normal cognitive function increased to 40%. Prior to cochlear implantation, 20% had abnormal cognitive scores on three of six cognitive tests, but this declined to 5% post-implantation. Implantation also resulted in improved speech perception, enhanced quality of life, and decreased depression. There was a strong relationship between scores on long-term memory and speech in noise possibly due to the fact that working memory is important for understanding speech in noise (Javanbakht et al., 2021).

Hearing aids assist individuals with moderate hearing loss to understand speech in quiet and noise by reducing the cognitive load (Glick and Sharma, 2020) and improving communication in social interactions. However, it is unclear whether hearing aids prevent cognitive decline. Some have found that hearing aids provide no benefits (Dawes et al., 2015) while others have reported positive results. In an experimental trial of adults with mid-moderate hearing loss, 6 months use of hearing aids improved global cognitive function, executive function, visual working memory, and increased cognitive processing speed (Glick and Sharma, 2020). Evoked potential measurements indicated that these improvements were correlated with restoration of more normal cortical. Over a 25-year longitudinal study, use of a hearing aid among individuals with self-reported hearing loss slowed cognitive decline (Amieva et al., 2015). Other reports indicate that hearing aids improve executive function and working memory with greater benefit for females than males (Sarant et al., 2020). The improved speech intelligibility in noise that hearing aids provide would be expected to enhance short-term working memory (Rudner et al., 2012; Neher et al., 2018), but it is unclear if hearing aids enhance long-term memory given that hearing loss is more detrimental to long-term than short-term memory (Rönnberg et al., 2011; Ng et al., 2014). Only 20% of individuals that would benefit from a hearing aid actually own one (Chien and Lin, 2012). Thus, increasing the acceptance of hearing aids among potential beneficiaries represents a significant opportunity for improving both hearing as well as better brain health.

### Physical Activity, Neurogenesis, Memory, and Cognition

Although there is considerable interest in identifying pharmacological interventions to prevent dementia and AD, life style changes in the form of increased physical activity may offer significant benefit. Exercise greatly enhance neurogenesis, learning and memory in animal models (van Praag et al., 1999a, b), effects associated with increased expression of brain derived neurotrophic factor (BDNF) in the hippocampus (Adlard et al., 2004; Okamoto et al., 2021) and decreased amyloid protein levels in transgenic AD mice (Adlard et al., 2005). Epidemiological studies suggest that physical activity and fitness significantly reduces cognitive decline and AD (Laurin et al., 2001; Lytle et al., 2004; Podewils et al., 2005; Ross et al., 2016). These benefits may be mediated by various molecules released during physical exercise (Tari et al., 2019) such as BDNF which enhances learning, and prevents cognitive decline (Cotman and Berchtold, 2002; Cotman and Engesser-Cesar, 2002). Physical exercise also upregulates insulin-like growth factor-1 (IGF-1), a neuroprotective molecule. Low levels of IGF-1 are associated with AD whereas high levels are linked to increased hippocampal volume (Westwood et al., 2014) and enhanced learning and memory (Cetinkaya et al., 2013). IGF-1 also promotes hippocampal cell proliferation that had been depressed by prior cisplatin treatment (Janelsins et al., 2010). These observations are consistent with several epidemiological studies indicating that physical activity protects against cognitive decline and AD (Laurin et al., 2001; Sofi et al., 2011). Exercise also slows the progression of hearing loss in animal models of presbycusis (Han et al., 2016) consistent with epidemiological studies (Gispen et al., 2014; Haas et al., 2016; Kawakami et al., 2022). In an increasingly sedentary world, a consistent moderate, daily dose of physical exercise may promote better hearing and brain health.

## Limitations

The animal studies discussed in this review indicate that chronic or acute noise exposure can suppress hippocampal neurogenesis and impair spatial learning and memory, but further work is needed to address a number of important questions. In cases of significant permanent NIHL, the literature indicates that these deficits persist up to 3–4 months post-exposure, but longer duration studies are needed to determine if these deficits continue or improve with longer recovery times. If the deficits get worse over time, then it would be important to evaluate potential mechanisms that contribute to this decline and to identify therapeutic interventions to prevent this.

The noise-induced disturbances in place cell function (Figure 3) represents an acute effect of an acute noise exposure that would likely only cause a temporary hearing loss. Future studies are needed to determine if more severe noise exposures cause permanent disruption of hippocampal place cell function. While the acute noise-induced changes in place cell function suggest that they could contribute to permanent disturbances in spatial navigation and memory impairment, we are unaware of any studies in which the noise-induced functional changes in place cell function have been correlated with a long-term, persistent decline in neurogenesis or long-term deficits on spatial memory acquisition or memory retention. Future studies aimed at investigating the relationships between hippocampal place cell dysfunction and neurogenesis and the relationship between place cell dysfunction and spatial memory deficits represent promising areas of future research.

Because many urban environments are characterized by moderate intensity intermittent and unpredictable noise exposures that cause little threshold shift, it would be important to determine if prolonged exposure to such noise permanently disrupts neurogenesis, learning and memory. Indeed, there is growing interest in noise-exposure that cause little threshold shift due to synaptopathy that reduce the flow of auditory information to the central nervous system (Kujawa and Liberman, 2015; Shi et al., 2016). Well-controlled animal studies could evaluate this. Intense exposures that induce permanent NIHL increase GR expression in the hippocampus. The chronic upregulation of hippocampal GR expression would presumably disrupt negative feedback in the HPA axis. Because GRs are ubiquitously expressed throughout the central nervous system, it could be useful to determine if GRs are up or downregulated elsewhere in the brain. This review tended to focus on noise-induced stress as a major factor in suppressing hippocampal neurogenesis, but because conductive hearing loss can suppress neurogenesis (Zhao et al., 2018; Kurioka et al., 2021), auditory deprivation, independent of stress may be sufficient to suppress hippocampal neurogenesis. To test the role of auditory deprivation as a major factor in suppressing hippocampal neurogenesis independent of stress, it would be important to determine if stress hormones and stress hormone receptors change or remain stable following auditory deprivation.

An important clinical question is whether the noise-induced disruptions to neurogenesis, learning and memory can be reversed by increased physical activity, an enriched environment or pharmacologic interventions and if so, what is the optimal time to do so. These and other related questions provide a framework that could be addressed in future preclinical studies.

## Author Contributions

SM, DD, and G-DC: conceptualization, visualization, and writing. LL, JW, Y-CC, and LC: conceptualization and writing. RS: conceptualization, visualization, writing, and funding acquisition. All authors contributed to the article and approved the submitted version.

## Conflict of Interest

The authors declare that the research was conducted in the absence of any commercial or financial relationships that could be construed as a potential conflict of interest.

## Publisher’s Note

All claims expressed in this article are solely those of the authors and do not necessarily represent those of their affiliated organizations, or those of the publisher, the editors and the reviewers. Any product that may be evaluated in this article, or claim that may be made by its manufacturer, is not guaranteed or endorsed by the publisher.
